# Supplementary material for: Twin-arginine transport complex plays an essential role in Caulobacter cell shape and viability
Source: Mol Biol Cell. 2026 Feb 13;37(3):br8. doi: 10.1091/mbc.E24-12-0578 (PMC12911857; doi:10.1091/mbc.E24-12-0578)
Supplement: Supplementary file 4 [file reviewer_reportE24-12-0578.pdf]

Twin-arginine transport complex plays an essential role in Caulobacter cell shape and viability

Trisha Chong, Klara Christensen, Damion Whitfield, Mayura Panjalingam, Nima Pendar, Phway Phway Myat, and Joseph Chen

Corresponding author(s): Joseph Chen, San Francisco State University

Review Timeline:

Submission Date:	2024-12-24
Editorial Decision:	2025-01-21
Revision Received:	2025-10-31
Editorial Decision:	2025-12-09
Revision Received:	2025-12-25
Accepted:	2026-01-02

Editor-in-Chief: John Aitchison

Transaction Report:

(Note: With the exception of the correction of typographical or spelling errors that could be a source of ambiguity, letters and reports are not edited. The original formatting of letters and referee reports may not be reflected in this compilation.)

RE: Manuscript #E24-12-0578
TITLE: Twin-arginine transport complex plays an essential role in Caulobacter cell shape and viability

Dear Dr. Chen:

Thanks for your submission. You'll see that both reviewers feel your manuscript begins with interesting observations, but that the conclusions are not fully supported by the data presented. They suggest substantial additional experiments to support the conclusions. If you are able to respond to their concerns, including showing stronger evidence for role of Tat-mediated secretion of PBP2 in the essentiality of Tat, we can consider a revised manuscript. If you do submit a revised manuscript, please refer to the MBoC author checklist and ensure that your manuscript adheres to the guidelines outlined there.

Sincerely,

Erin Goley
Monitoring Editor
Molecular Biology of the Cell

Dear Dr. Chen,

The review of your manuscript, referenced above, is now complete. The Monitoring Editor has decided that your manuscript is not acceptable for publication at this time, but may be deemed acceptable after specific revisions are made, as described in the Monitoring Editor's decision letter above and the reviewer comments below.

A reminder: Please do not contact the Monitoring Editor directly regarding your manuscript. If you have any questions regarding the review process or the decision, please contact the MBoC Editorial Office (mboc@ascb.org).

When submitting your revision include a rebuttal letter that details, point-by-point, how the Monitoring Editor's and reviewers' comments have been addressed. (The file type for this letter must be "rebuttal letter"; do not include your response to the Monitoring Editor and reviewers in a "cover letter.") Please bear in mind that your rebuttal letter will be published with your paper if it is accepted, unless you have opted out of publishing the review history.

Authors are allowed 90 days to submit a revision. If this time period is inadequate, please contact us at mboc@ascb.org.

Revised manuscripts are assigned to the original Monitoring Editor whenever possible. However, special circumstances may preclude this. Also, revised manuscripts are often sent out for re-review, usually to the original reviewers when possible. The Monitoring Editor may solicit additional reviews if it is deemed necessary to render a completely informed decision.

In preparing your revised manuscript, please follow the instruction in the Information for Authors (www.molbiolcell.org/info-for-authors). In particular, to prepare for the possible acceptance of your revised manuscript, submit final, publication-quality figures with your revision as described.

To submit the rebuttal letter, revised manuscript, and figures, use this link: Link Not Available

MBoC offers the option to publish your work with immediate open access. Open access can increase the discoverability and usability of your research. If you would like to publish your paper with immediate open access but did not select this option during initial submission, please contact the editorial office at mbc@ascb.org.

Authors of Articles and Brief Communications are also encouraged to create a short video abstract to accompany their article when it is published. These video abstracts, known as Science Sketches, are up to 2 minutes long and will be published on YouTube and then embedded in the article abstract. Science Sketch Editors on the MBoC Editorial Board will guide you as you prepare your video. Information about how to prepare and submit a video abstract is available at www.molbiolcell.org/science-sketches. Please contact mboc@ascb.org if you are interested in creating a Science Sketch.

Sign up for MBoC's new content alerts here https://tinyurl.com/3bcsyk3e

Thank you for submitting your manuscript to MBoC. We look forward to receiving your revised paper.

Sincerely,

Eric Baker
Journal Production Manager
MBoC Editorial Office
mbc@ascb.org

Reviewer #1 (Remarks to the Author):

Twin-arginine transport complex play an essential role in Caulobacter cell shape and viability
TN Chong, DL Whitfield, M Panjalingam, N Pendar, JC Chen

Summary
The authors present a somewhat compelling demonstration that the twin-arginine translocation pathway (TAT) is potentially essential in Caulobacter crescentus. The essentiality is confirmed via plasmid loss assay and depletion strain phenotypes. The authors suggest that the essentiality of TAT is due at least in part to a role in localizing PBP2. This claim is supported by localization studies; however, the difference in phenotypes caused TAT and PBP2 depletion suggest that there is more to this (as eluded to by the authors in the discussion).

Several claims in the abstract and significance statement are largely unsupported by the data provided. In particular, the third claim in the significance statement that the interaction between the Tat complex and Pbp2 are contributing to viability and maintenance of cell shape are not supported. No interaction assessments were performed (experimentally or bioinformatically) and the discussion mentions the possibility of indirect effects of TAT on PBP2 localization via other substrates.

Major Comments:
1. There are several descriptive errors throughout the introduction and discussion sections of this manuscript that need to be corrected for accuracy. Examples include:
- Lines 91-98: descriptions of MreB and associated proteins forming spirals reads as though these are static spirals and does not reflect the current view of this dynamic polymer
- PBP2 is described erroneously as a cytoskeletal protein (line 150-151) and a PG remodeling protein (lines 203-204)) while also be described as essential for elongation (lines 86-88). Description of PBP2 function should be consistent.

2. Expression levels of key proteins explored in this study are not compared across strains or conditions. The observed shifts in localization patterns could be influenced by undetected changes in protein stability or abundance.

3. Figures 1 and S1 and related text -It looks like there is a great deal of lysis in the tat mutants. The phenotypic difference between the tat mutants is not sufficiently detailed. Consider including the Figure S1 content in the main figure to allow comparisons across strains. The "similarity" between the tat and pbp2 strains is not particularly clear from the few cells shown. Can the morphological changes be quantified to support this claim? Is it possible that suppressors are arising in the populations accounting for the variance in growth in the tatA, tatC, and pbp2 mutants?

4. Lines 167-169 and Expression strains panel of Figure 1 B. Consider moving this supplemental.

5. Figure S1 legend - the legend states that similar results were obtained with CB-15 derived strains. The data should be included or the comment removed. If no data from the CB-15 derived strains is included, these strains and methods should be removed from the manuscript.

6. For the localization studies of PBP2 and MreB only localization in the tatA depletion is shown. Is it possible that the remaining Tat components may have differential impact on localization? These seems to be an important consideration given the different subcellular locations of the targets (periplasmic and cytoplasmic) and the different topologies of the TAT proteins along with the phenotypic variation of the mutants.

7. Figures 2 and 3 - Since quantitative data are presented, statistical assessment of difference between populations should be completed. Why is the mid-line and regions meeting a 0.75 threshold shown only in Figure 3 and not Figure 2?

Minor Comments
1. Line 77 -78. The transition here in the introduction from suggesting that one or more TAT substrates may contribute to essentiality to introductory content about PG is sharp. Consider using the fact that PBP2 is predicted to be a TAT substrate (as detailed in lines 211-214) as a rationale for considering the impact of TAT secretion on PBP2 and cell wall synthesis.

2. Line 147 - "some cells seem to have ruptured". It looks like there is a great deal of lysis

3. Line 153: Explain the rationale for why only the transpeptidase domain of PBP1a is deleted?

4. Lines 167-169 and Expression strains panel of Figure 1 B. Consider moving this supplemental.

5. Figure S1 - add delta symbols and italicize for the tat mutant labels.

6. Figure 2 - Panels A and B are not scaled the same. Are Cc cells on M2G pads truly up to 8 um long? Or is the distance from mid-cell label incorrect?

7. Figures 2 and 3 - Schematics showing axis of quantification are helpful but not described in the figure legend.

8. Given that this is combined results and discussion section it would be helpful to integrate the discussion throughout rather than just in the final section.

Reviewer #2 (Remarks to the Author):

In their Brief Report manuscript, "Twin-arginine transport complex plays an essential role in Caulobacter cell shape and viability," the authors Chong et al. investigate the essentiality of the Tat system in Caulobacter and its effect on morphology. They demonstrate that the depletion phenotypes of the Tat system and Pbp2 in Caulobacter are linked in producing similar morphological abnormalities, hypothesizing that this indicates that Pbp2 may be secreted by the Tat system. The authors further show that localization of Pbp2 to mid-cell during osmotic upshift depends on tatA using fluorescence microscopy experiments. They argue that because Pbp2 is not localized to the periphery (inner membrane) in the tatA depletion strain under normal growth conditions, it may itself be cargo for the Tat system, explaining the relationship between these proteins.

Overall, I found this paper to be interesting and of value to the cell biology field, however it over promises in its significance statements and does not provide enough data for a more definitive conclusion about the molecular relationship between the Tat system and Pbp2. I think that the authors demonstrate essentiality (or at least severe impairment of growth and morphology, see major concern 3) and loss of mid-cell localization of Pbp2 when tatA is depleted. However, by the end of the paper, it is still unclear whether the "interaction" the authors describe (line 32) is within a genetic network (with other players) or a direct physical interaction. The current data does not clearly indicate whether Pbp2 has been correctly secreted in the tatA depletion, which is an interesting question. I think that this work is promising but lacks a few experiments that would make its conclusions distinct.

Major concerns (listed in order of importance, not in order of occurrence in MS):
1. Fig. 3A intends to show that "TatA I is essential for Pbp2 localization to the cell periphery" (figure legend title).
a. This is already a very vague statement - the authors do not indicate that they interpret this to mean secretion to the inner membrane (which would be helpful for a general cell-molecular audience). The authors should clarify their logic better -- if there is a third component that component that recruits Pbp2 to mid-cell under osmotic upshifts, its secretion into the IM probably would not impact Pbp2 localization to the periphery under normal conditions. The authors do mention this shortcoming (lines 214-216) but I think publication of this MS in MBoC requires a bit more clarity.
b. The images do not appear to be of high enough quality here to make this vague statement, although I understand that the quantification is included to validate it. This result is suggestive but not definitive. These images and analysis should be accompanied by one of the following to support and distinguish these outcomes:
i. You use better imaging that can show increased (not just extended) fluorescence intensity in the cell periphery.
ii. You use, as a control, a known Tat system cargo that demonstrates the same behavior +/- tatA.
iii. You show that removing the twin-arginine motif in the pbp2 sequence results in the same localization pattern.
iv. You show that Pbp2 is detected at lower levels in the periplasm during a tatA depletion using a complementary method (ex. x-linking to PG and then detecting with Western blot or other assay).
2. This MS does not correctly contextualize this study within a modern body of research that would make it an even more interesting paper and possibly inform better experimentation. In lines 91-96, bacteria are described as having a "spiral shaped" cytoskeleton. This was an early theory but these spirals were shown to be an artifact of the MreB fluorescent protein fusion reporter and is no longer accepted by the field. MreB is a cytoskeletal element that forms dynamic, short, antiparallel filaments associated with the membrane that are dispersed along the long axis of the rod-shaped bacterium. The movement of these filaments are driven by enzymatic activity of the complex MreB forms with other components of the Rod system, the enzymatic core of which is RodA and Pbp2. The authors should rewrite the introduction to revise this information to better indicate the relationship of Pbp2 and MreB within the Rod system. This context is especially noteworthy because Hocking et al. 2012 (which your work follows up on) indicates that RodA and PBP1a also localized to mid-cell upon osmotic upshift along with Pbp2. This information leads to a few suggestions for analysis ad experimentation that would improve this manuscript:
a. Do any other components of the Rod system have twin-arginine signal peptide in Caulobacter?
b. Is the localization of RodA or PBP1 to mid-cell during osmotic upshift influenced by the tatA depletion?
Exploration of dependence of RodA or PBP1 on Pbp2 and/or the Tat system could clarify major standing questions about the elongasome in Caulobacter and how it is regulated under stress.
3. It is interesting that in Figure 1B, depletion of each of the Tat components exhibit different levels of impairment of growth and

morphology. Arguably, depletion of tatA and tatC have severe fitness defects whereas tatB is essential. Has this been seen for other Tat systems? A strain for depleting the entire locus tatABC was tested in Figure S1... are the phenotypes additive for this strain? This is an interesting discussion point on the side of the broader community seeking to understand the essentiality of the Tat system and the roles of its various components. Why was tatA chosen as the genetic background for the experiments in Figure 2 and 3?
4. Biological replicates for the experiments have not been discussed if they were done.

Some minor points:

1. The MS makes the point that the Tat apparatus is dispensable for viability in "most" bacteria (lines 41-42, 70-71, 106) with no support from primary literature, just a reference to a review. Considering that essentiality must be empirically proven and that less than 1% of bacteria have even been cultured, I would recommend revising language on this, especially since the species shown to require the Tat system are spread throughout the domain. It is remarkable that the model bacterium E. coli does not require it, and perhaps this investigation in Caulobacter can help us to understand variation in the essentiality of the Tat system.
2. E. coli encodes more Tat system components - TatABCDE - does Caulobacter only have TatABC (line75) or were these the only components identified as essential in the Tn-seq screen?
3. In lines 89-103 it is hard to know whether you are describing Pbp2 in E. coli or Caulobacter without looking up the references. It would be helpful to write more clearly in this section about what is known for Caulobacter vs. E. coli.
4. Lines 125-133 describe the information shown in Figure 1A in the opposite order that they appear in the figure. I would make them match so that it is less confusing.
5. Line 142 reports the average final OD610 of TatB to be 0.17 +/-0.01. In Figure 1B, it appears to be well below 0.05. Is there an explanation for this?
6. Line 151: Pbp2 is not a cytoskeletal protein; it is a PG-modifying periplasmic enzyme.
7. Line 153-154: since your pbp2 depletion strain does not deplete the entirety of Pbp2, just its transpeptidase domain, I would consider clarifying this in the notation for this strain in the figures and text.
8. In Figure 1, "C" is not labeled. I would consider breaking down these multiple components into i/ii/iii, etc, so you can discuss them more clearly in the text.
9. It could be helpful to do quantitative analysis on the morphologies of the different Tat and Pbp2 depletions using MicrobeJ (which you used in Fig 2 and 3 for demographs). This would help illustrate the degree to which the phenotypes differ.
10. The quantification in Figure 2AB is never discussed in the MS.
11. Line 192 says the cells were grown in in M2G and immobilized on M2G pads whereas Figure 3 says "PYE culture -> PYE pad."

Dear Dr. Goley:

We thank the reviewers for their thoughtful suggestions and insightful feedback on how to improve the manuscript. We appreciate the patience of the MBoC Editorial Office while we responded to all the reviewers' comments by performing additional experiments and revising the manuscript extensively. Each of the reviewers' remarks is addressed below. The text of the original decision letter is shown in black Times New Roman font, while our responses are in blue Arial font.

We believe that the modifications have improved the manuscript substantially and hope that it now merits publication in *Molecular Biology of the Cell*.

Sincerely,
Joseph Chen

Dear Dr. Chen:

Thanks for your submission. You'll see that both reviewers feel your manuscript begins with interesting observations, but that the conclusions are not fully supported by the data presented. They suggest substantial additional experiments to support the conclusions. If you are able to respond to their concerns, including showing stronger evidence for role of Tat-mediated secretion of PBP2 in the essentiality of Tat, we can consider a revised manuscript. If you do submit a revised manuscript, please refer to the MBoC author checklist and ensure that your manuscript adheres to the guidelines outlined there.

Sincerely,

Erin Goley
Monitoring Editor
Molecular Biology of the Cell

Reviewer #1 (Remarks to the Author):

Twin-arginine transport complex play an essential role in Caulobacter cell shape and viability
TN Chong, DL Whitfield, M Panjalingam, N Pendar, JC Chen

Summary
The authors present a somewhat compelling demonstration that the twin-arginine translocation pathway (TAT) is potentially essential in Caulobacter crescentus. The essentiality is confirmed

via plasmid loss assay and depletion strain phenotypes. The authors suggest that the essentiality of TAT is due at least in part to a role in localizing PBP2. This claim is supported by localization studies; however, the difference in phenotypes caused TAT and PBP2 depletion suggest that there is more to this (as eluded to by the authors in the discussion).

Several claims in the abstract and significance statement are largely unsupported by the data provided. In particular, the third claim in the significance statement that the interaction between the Tat complex and PBP2 are contributing to viability and maintenance of cell shape are not supported. No interaction assessments were performed (experimentally or bioinformatically) and the discussion mentions the possibility of indirect effects of TAT on PBP2 localization via other substrates.

We appreciate this thoughtful comment. Based on the available evidence, the interaction that we intended in the original manuscript is a genetic one. We agree that additional work would be needed to demonstrate biochemically that PBP2 interacts directly with the Tat complex, but that is beyond the scope of this study.

We have updated the third significance statement in the revised manuscript to address this concern (lines 33-34): "PBP2 is critical for viability and maintenance of cell shape in *Caulobacter*, and essentiality of the Tat complex may be partly attributed to a role in localizing PBP2."

Major Comments:

1. There are several descriptive errors throughout the introduction and discussion sections of this manuscript that need to be corrected for accuracy. Examples include:
- Lines 91-98: descriptions of MreB and associated proteins forming spirals reads as though these are static spirals and does not reflect the current view of this dynamic polymer
- PBP2 is described erroneously as a cytoskeletal protein (line 150-151) and a PG remodeling protein (lines 203-204)) while also be described as essential for elongation (lines 86-88). Description of PBP2 function should be consistent.

We thank the reviewer for this correction. We have revised the text to refer to PBP2 as a transpeptidase throughout the text. We have also changed the Introduction to describe PBP2 as a transpeptidase in the Rod complex and provided additional references to reflect an updated view of that complex's interaction with MreBCD (lines 94-99):

"Main components of the Rod complex include the PG polymerase RodA and the transpeptidase PBP2, which associate with the actin-like cytoskeletal protein MreB and multiple other proteins, including MreC, MreD, RodZ, and PBP1a; the cytoskeletal elements MreBCD form a dynamic scaffold that guides peptidoglycan synthesis by RodA and PBP2 (Banzhaf *et al.*, 2012; Bratton *et al.*, 2018; Liu *et al.*, 2020; Morgenstein *et al.*, 2015; Özbaykal *et al.*, 2020; Rohs *et al.*, 2018)."

2. Expression levels of key proteins explored in this study are not compared across strains or conditions. The observed shifts in localization patterns could be influenced by undetected changes in protein stability or abundance.

To address this concern, we have performed immunoblot analysis to assess steady-state levels of mCherry-PBP2 in TatA and TatC depletion strains and of ssTorA-dimer2 (fusion of a canonical Tat signal sequence to the dimer2 fluorescent protein) in the TatA depletion strain (Figure 3, C and F). Levels of mCherry-PBP2 were comparable between cells replete with or depleted of TatA or TatC, while ssTorA-dimer2 showed accumulation of its unprocessed form in TatA-depleted cells, indicative of a translocation defect. We describe and discuss these results in the text (lines 290-298, 325-332).

3. Figures 1 and S1 and related text -It looks like there is a great deal of lysis in the tat mutants. The phenotypic difference between the tat mutants is not sufficiently detailed. Consider including the Figure S1 content in the main figure to allow comparisons across strains. The "similarity" between the tat and pbp2 strains is not particularly clear from the few cells shown. Can the morphological changes be quantified to support this claim? Is it possible that suppressors are arising in the populations accounting for the variance in growth in the tatA, tatC, and pbp2 mutants?

We thank the reviewer for these insights. Yes, many of the cells lysed when grown under Tat depletion conditions. We have added estimates of this lysis in lines 157-159: “significant fractions of cells appeared ruptured, ranging from approximately 30% in Δ*tatB* to 90% in Δ*tatA* and Δ*tatC* strains (Figure 1C).” Moreover, we now report morphological analysis of cell “roundness,” as calculated by MicrobeJ (Figure 1D), showing that roundness increases when TatA, TatB, TatC, or PBP2 is depleted. We also discuss possible explanations for variations in the severity of morphological changes among Tat and PBP2 depletion strains in the text (lines 198-204). That is in addition to the possibility that differences in the expression and cellular requirements of individual proteins may lead to variations in the timing and extent of growth arrests (lines 144-150).

While cells were taken from frozen stocks before experiments, we cannot rule out that suppressor mutations emerged during re-streaking, growth in liquid cultures, or strain construction. We typically characterized at least two isolates of each constructed strain and observed similar phenotypes for different isolates. (For simplicity, we usually chose one for more extensive analysis). However, strong selective pressure may have led to inevitable appearance of suppressors. Nevertheless, repeated trials did indicate that the strains behave in consistent ways. For example, the TatC depletion strain took longer than the TatA depletion strain to show growth arrest in liquid cultures (Figure 1B) and to exhibit mislocalization of mCherry-PBP2 (lines 250-254).

At this point, we have not investigated the rates of suppressor mutations but do mention the possibility of suppressors in the text specifically for the TatB depletion strain, as PBP2 does not appear to localize normally in that strain even when *tatB* is induced (lines 236-249). Identification of mutations that can alleviate the loss of Tat activity may be an intriguing pursuit in the future.

4. Lines 167-169 and Expression strains panel of Figure 1 B. Consider moving this supplemental.

We have followed this advice and rearranged Figure 1 accordingly. Phase contrast micrographs of Tat and PBP2 expression strains are now in Figure S1B. This rearrangement allowed us to show comparisons of the roundness measurements in Figure 1D.

5. Figure S1 legend - the legend states that similar results were obtained with CB-15 derived strains. The data should be included or the comment removed. If no data from the CB-15 derived strains is included, these strains and methods should be removed from the manuscript.

We understand this concern and have now included colony photos of CB15-derived strains in Figure S1A.

6. For the localization studies of PBP2 and MreB only localization in the tatA depletion is shown. Is it possible that the remaining Tat components may have differential impact on localization? These seems to be an important consideration given the different subcellular locations of the targets (periplasmic and cytoplasmic) and the different topologies of the TAT proteins along with the phenotypic variation of the mutants.

We have now assessed mCherry-PBP2 localization in TatB and TatC depletion strains (Figure S3, A-D). The results for TatC depletion resemble those of TatA depletion. For the TatB depletion strain, mCherry-PBP2 localized poorly to the midcell after osmotic shock, even when TatB should be replete. We address these results in the updated manuscript (lines 236-261). Because depletion of TatC requires more time than depletion of TatA, we performed most experiments with the TatA depletion strain.

7. Figures 2 and 3 - Since quantitative data are presented, statistical assessment of difference between populations should be completed. Why is the mid-line and regions meeting a 0.75 threshold shown only in Figure 3 and not Figure 2?

We appreciate this suggestion. We have modified how the fluorescence images were analyzed to provide statistical assessment of differences between populations. We have now included area under curve (AUC) as a way to compare normalized fluorescence profiles along the medial axis (Figure 2, Figure S3). We use normalized fluorescence at the midpoint of the cell for comparisons along the transverse axis (Figure 3, Figure S4). Different approaches were used for the two axes because the localization signals and fluorescence profiles were qualitatively distinct between experiments with or without osmotic upshift.

We describe these statistical comparisons first in the Results and Discussion section (such as lines 230-234, 283-290) and with more details in the Materials and Methods section (lines 424-439).

Minor Comments

1. Line 77 -78. The transition here in the introduction from suggesting that one or more TAT substrates may contribute to essentiality to introductory content about PG is sharp. Consider using the fact that PBP2 is predicted to be a TAT substrate (as detailed in lines 211-214) as a rationale for considering the impact of TAT secretion on PBP2 and cell wall synthesis.

We thank the reviewer for this suggestion. We agree that the transition was ineffective. We have now tried to create a smoother transition by mentioning that some known substrates of the Tat pathway are involved in cell wall maintenance (lines 85-88):

"Various substrates of the Tat pathway in *E. coli*, such as the AmiA and AmiC amidases, and in other bacteria participate in cell wall maintenance and metabolism (Bernhardt & de Boer, 2003; Brauer *et al.*, 2021; Gallego-Parrilla *et al.*, 2024; Ize *et al.*, 2003; Saint-Joanis *et al.*, 2006)."

We then discuss briefly the role of the Rod complex (lines 92-99) and also state that PBP2 is a potential Tat substrate in *Caulobacter* (lines 103-105). We mention AmiA and AmiC to allude to the fact that the Tat machinery, while not essential for viability in *E. coli*, does export proteins involved in cell wall maintenance in that system.

2. Line 147 - "some cells seem to have ruptured". It looks like there is a great deal of lysis

Yes, many cells lysed under Tat depletion conditions. We have added estimates of cell lysis to the text (lines 157-159): "significant fractions of cells appeared ruptured, ranging from approximately 30% in Δ*tatB* to 90% in Δ*tatA* and Δ*tatC* strains (Figure 1C)." These estimates are based on visual inspection of multiple images, which is challenging due to the abundance of cell debris.

3. Line 153: Explain the rationale for why only the transpeptidase domain of PBP1a is deleted?

(We assume that the reviewer meant PBP2 instead of PBP1a.) The region encoding full-length PBP2 could not be deleted, likely due to polar effects. We address this possibility in the revised manuscript (lines 167-170):

"As in another study (Wagner *et al.*, 2005), we were unable to generate a deletion of the complete *pbp2* coding sequence, even while expressing it from the P_{xyl} promoter, possibly due to polar effects on the essential *rodA* gene downstream (Christen *et al.*, 2011)."

4. Lines 167-169 and Expression strains panel of Figure 1 B. Consider moving this supplemental.

We appreciate this suggestion. As indicated above (in the response to Major Comment #4), we have moved the phase contrast images for expression strains to Figure S1B.

5. Figure S1 - add delta symbols and italicize for the tat mutant labels.

For Figure S1A, we have now added Δ symbols to the appropriate column headings to denote which strains had deletion mutations and have also italicized gene names in the row headings.

6. Figure 2 - Panels A and B are not scaled the same. Are Cc cells on M2G pads truly up to 8 um long? Or is the distance from mid-cell label incorrect?

We have changed the images and graphs in Figures 2 and S3 in the revised manuscript. In particular, we have re-scaled the demographs to be from -2 to 2 μm long. Previous demographs that were -4 to 4 μm included a gap on either side of the cells.

7. Figures 2 and 3 - Schematics showing axis of quantification are helpful but not described in the figure legend.

We thank the reviewer for pointing out this oversight. We have included descriptions of the schematics in the figure legends for Figures 2, 3, S3, and S4. Below is an example of the description for Figure 2, B and D (lines 520-522):

"Medial profiles (left panels) represent normalized fluorescence intensities along normalized cell length, as illustrated by the schematic of a model cell on the left."

8. Given that this is combined results and discussion section it would be helpful to integrate the discussion throughout rather than just in the final section.

We appreciate this suggestion. We have integrated more discussion throughout the Results and Discussion section in the revised manuscript. For example, we discuss possible explanations for differences in phenotype severity between Tat and PBP2 depletion strains in lines 198-209.

Reviewer #2 (Remarks to the Author):

In their Brief Report manuscript, "Twin-arginine transport complex plays an essential role in

Caulobacter cell shape and viability," the authors Chong et al. investigate the essentiality of the Tat system in Caulobacter and its effect on morphology. They demonstrate that the depletion phenotypes of the Tat system and Pbp2 in Caulobacter are linked in producing similar morphological abnormalities, hypothesizing that this indicates that Pbp2 may be secreted by the Tat system. The authors further show that localization of Pbp2 to mid-cell during osmotic upshift depends on tatA using fluorescence microscopy experiments. They argue that because Pbp2 is not localized to the periphery (inner membrane) in the tatA depletion strain under normal growth conditions, it may itself be cargo for the Tat system, explaining the relationship between these proteins.

Overall, I found this paper to be interesting and of value to the cell biology field, however it over promises in its significance statements and does not provide enough data for a more definitive conclusion about the molecular relationship between the Tat system and Pbp2. I think that the authors demonstrate essentiality (or at least severe impairment of growth and morphology, see major concern 3) and loss of mid-cell localization of Pbp2 when tatA is depleted. However, by the end of the paper, it is still unclear whether the "interaction" the authors describe (line 32) is within a genetic network (with other players) or a direct physical interaction. The current data does not clearly indicate whether Pbp2 has been correctly secreted in the tatA depletion, which is an interesting question. I think that this work is promising but lacks a few experiments that would make its conclusions distinct.

We appreciate the reviewer's insights and have performed additional experiments and analyses to make the conclusions more concrete. We intended to indicate a genetic interaction between the Tat complex and PBP2 in the original manuscript but have now altered the significance statement in response to both reviewers' comments (lines 33-34): "PBP2 is critical for viability and maintenance of cell shape in *Caulobacter*, and essentiality of the Tat complex may be partly attributed to a role in localizing PBP2."

Major concerns (listed in order of importance, not in order of occurrence in MS):

1. Fig. 3A intends to show that "TatA I is essential for Pbp2 localization to the cell periphery" (figure legend title).
a. This is already a very vague statement - the authors do not indicate that they interpret this to mean secretion to the inner membrane (which would be helpful for a general cell-molecular audience). The authors should clarify their logic better -- if there is a third component that recruits Pbp2 to mid-cell under osmotic upshifts, its secretion into the IM probably would not impact Pbp2 localization to the periphery under normal conditions. The authors do mention this shortcoming (lines 214-216) but I think publication of this MS in MBoC requires a bit more clarity.

We have followed these suggestions. We have modified the Figure 3 legend title to "TatA is essential for targeting mCherry-PBP2 and ssTorA-dimer2 to the cell envelope" (lines 531-532). This title accommodates both insertion of PBP2 into the inner membrane and export of ssTorA-dimer (a fusion protein with an *E. coli* Tat signal sequence previously used in a *Caulobacter* study) into the periplasm.

In addition, we now include an explanation that localization to the cell periphery is consistent with membrane association (lines 280-287):

"We observed that mCherry-PBP2 localized predominantly to the cell periphery in WT and TatA-replete cells, consistent with insertion into the membrane; in contrast, TatA-depleted cells grown with glucose exhibited fluorescence throughout the cell body (Figure 3A). Plots of normalized fluorescence profiles along the transverse axis (across the cell width) of WT and TatA-replete cells displayed two peaks, corresponding to localization to the cell membrane on either side of the cell, whereas TatA-depleted cells showed a single central peak, suggesting cytosolic localization (Figure 3B)."

We also provide references to indicate that alternative explanations are possible for improper translocation of PBP2 in the absence of the Tat complex (lines 357-361):

"Although direct translocation is the simplest explanation, we cannot rule out that PBP2 translocation may involve cooperation between the Tat and Sec pathways (Keller *et al.*, 2012; Tooke *et al.*, 2017) or a hitchhiking mechanism with another Tat substrate (Gallego-Parrilla *et al.*, 2024; Rodrigue *et al.*, 1999)."

b. The images do not appear to be of high enough quality here to make this vague statement, although I understand that the quantification is included to validate it. This result is suggestive but not definitive. These images and analysis should be accompanied by one of the following to support and distinguish these outcomes:
i. You use better imaging that can show increased (not just extended) fluorescence intensity in the cell periphery.
ii. You use, as a control, a known Tat system cargo that demonstrates the same behavior +/- tatA.
iii. You show that removing the twin-arginine motif in the pbp2 sequence results in the same localization pattern.
iv. You show that Pbp2 is detected at lower levels in the periplasm during a tatA depletion using a complementary method (ex. x-linking to PG and then detecting with Western blot or other assay).

We agree that additional work was needed to show more definitively that PBP2 relies on the Tat system for export. To address this concern, we have followed suggestion (ii) of the reviewer: use a known Tat system cargo. We do not know of a native Tat substrate that has been empirically verified in *Caulobacter*; thus, we used a fusion between dimer2 and the canonical *E. coli* Tat signal sequence from TorA, previously demonstrated to target to the periplasm in *Caulobacter* (Judd *et al.*, 2005). We now show peripheral localization of this ssTorA-dimer2 fusion (Figure 3, D and E) (lines 320-323):

"As expected, WT and TatA-replete cells expressing the fusion exhibited fluorescence predominantly in their perimeters, while TatA-depleted cells displayed more diffuse fluorescence (Figure 3D), parallel to observations with mCherry-PBP2."

To quantify the difference between localization to the cell envelope and cytoplasm, we measured fluorescence profiles along the transverse axis and report the normalized fluorescent signal at the midpoint of the cell width, for both mCherry-PBP2 and ssTorA-dimer2 (Figure 3, B and E). We also provide better fluorescence images to demonstrate the differences in localization patterns more clearly (Figure 3, A and D).

2. This MS does not correctly contextualize this study within a modern body of research that would make it an even more interesting paper and possibly inform better experimentation. In lines 91-96, bacteria are described as having a "spiral shaped" cytoskeleton. This was an early theory but these spirals were shown to be an artifact of the MreB fluorescent protein fusion reporter and is no longer accepted by the field. MreB is a cytoskeletal element that forms dynamic, short, antiparallel filaments associated with the membrane that are dispersed along the long axis of the rod-shaped bacterium. The movement of these filaments are driven by enzymatic activity of the complex MreB forms with other components of the Rod system, the enzymatic core of which is RodA and Pbp2. The authors should rewrite the introduction to revise this information to better indicate the relationship of Pbp2 and MreB within the Rod system. This context is especially noteworthy because Hocking et al. 2012 (which your work follows up on) indicates that RodA and PBP1a also localized to mid-cell upon osmotic upshift along with Pbp2.

Following the advice of both reviewers, we have re-written this portion of the Introduction to be more inclusive of the broad body of literature relating to the Rod complex and its dynamic interaction with cytoskeletal elements (lines 91-99):

"Throughout growth and division, the PG layer must be remodeled to permit changes in cell shape (Sauvage *et al.*, 2008; Spratt, 1975). Studies in *E. coli* and other species indicated that PG remodeling during cell elongation is carried out by the Rod complex (Garde *et al.*, 2021). Main components of the Rod complex include the PG polymerase RodA and the transpeptidase PBP2, which associate with the actin-like cytoskeletal protein MreB and multiple other proteins, including MreC, MreD, RodZ, and PBP1a; the cytoskeletal elements MreBCD form a dynamic scaffold that guides peptidoglycan synthesis by RodA and PBP2 (Banzhaf *et al.*, 2012; Bratton *et al.*, 2018; Liu *et al.*, 2020; Morgenstein *et al.*, 2015; Özbaykal *et al.*, 2020; Rohs *et al.*, 2018)."

In the Results and Discussion section we now also include a possible, broader implication of our results (lines 351-354):

"Considering that the membrane anchor of PBP2 interfaces with RodA in *E. coli* (Liu *et al.*, 2020; Nygaard *et al.*, 2023; Shlosman *et al.*, 2023), how the constraints of that interaction are reconciled with recognition by the Tat machinery in alpha-proteobacteria remains to be elucidated."

This information leads to a few suggestions for analysis ad experimentation that would improve this manuscript:
a. Do any other components of the Rod system have twin-arginine signal peptide in Caulobacter?

We thank the reviewer for the thoughtful comment. We did not detect any Tat recognition signal after analyzing the protein sequences of other Rod complex components in *Caulobacter*. We now briefly mention this in the end of the Results and Discussion section (lines 365-367):

". . . many other proteins are predicted to be exported via the Tat pathway in *Caulobacter* (Dilks *et al.*, 2003). Aside from PBP2, they are not known to participate directly in cell wall synthesis . . ."

b. Is the localization of RodA or PBP1 to mid-cell during osmotic upshift influenced by the tatA depletion?
Exploration of dependence of RodA or PBP1 on Pbp2 and/or the Tat system could clarify major standing questions about the elongasome in Caulobacter and how it is regulated under stress.

We appreciate this suggestion and now report in the revised manuscript (lines 270-275) that mCherry-PBP1a still localizes to the midcell upon osmotic shock following TatA depletion (Figure S3, E and F). This result is consistent with *Caulobacter* PBP1a lacking a potential Tat signal peptide.

3. It is interesting that in Figure 1B, depletion of each of the Tat components exhibit different levels of impairment of growth and morphology. Arguably, depletion of tatA and tatC have severe fitness defects whereas tatB is essential. Has this been seen for other Tat systems? A strain for depleting the entire locus tatABC was tested in Figure S1... are the phenotypes additive for this strain? This is an interesting discussion point on the side of the broader community seeking to understand the essentiality of the Tat system and the roles of its various components. Why was tatA chosen as the genetic background for the experiments in Figure 2 and 3?

We thank the reviewer for pointing out these considerations. As all three components of the Tat complex were identified as essential in previous Tn-seq analyses of the *Caulobacter* genome (Christen *et al.*, 2011; Price *et al.*, 2018), we agree that it is interesting that they show distinct growth phenotypes. We now discuss why the phenotypes may differ among the Tat depletion strains (lines 144-150):

"Variations in the timing and extent of growth arrests among the distinct Tat depletion strains may be due to differences in expression and cellular requirements of individual proteins. For instance, the TatB depletion strain appeared to stop doubling more quickly than the other strains after inoculation into PYE with glucose, possibly because TatB expression from P_{xyl} is barely sufficient for normal operations, whereas TatA and TatC need more cell doublings for their protein levels to dip below the viable concentration requirements."

We also discuss possible explanations of why mCherry-PBP2 does not localize properly in the TatB depletion strain, even under inducing conditions (lines 241-249). Because the *tatB* background did not yield easily distinguishable results (Figure S3, A and B), and *tatA* allowed faster depletion than the *tatC* background (lines 250-255), we chose *tatA* as the main genetic background for experiments, as mentioned in the text (lines 258-261).

As indicated by the reviewer, there may be some redundancy between components of the Tat complex, specifically TatA and TatB, because TatA and TatB share structural similarity (Yen *et al.*, 2002), and many two-component Tat systems exist in Gram-positive bacteria with only TatA and TatC (Barnett *et al.*, 2008; Jongbloed *et al.*, 2004; Jongbloed *et al.*, 2006). Moreover, a minimal Tat translocase consisting solely of TatA and TatC allowed low-level translocation in *E. coli* (Blaudeck *et al.*, 2005), and TatA and TatB can occupy each other's binding site on TatC (Alcock *et al.*, 2016; Habersetzer

et al., 2017). While our results suggest that all three core components of the Tat pathway are essential in *Caulobacter*, we do not yet have sufficient details about the molecular operations of the Tat complex in our system to make definitive comparisons. The plasmid loss assay is qualitative at this point, and the depletion growth assays may not be directly comparable across different background, for instance due to different leakiness of expression from the xylose-regulated, complementing alleles. The unexpected behavior of mCherry-PBP2 in the TatB depletion strain also suggests that further investigation is required.

4. Biological replicates for the experiments have not been discussed if they were done.

Each experiment was done at least twice with biological replicates, and similar results were observed. We have included replicates for experiments in the Materials and Methods section and/or in the figure legends.

Some minor points:

1. The MS makes the point that the Tat apparatus is dispensable for viability in "most" bacteria (lines 41-42, 70-71, 106) with no support from primary literature, just a reference to a review. Considering that essentiality must be empirically proven and that less than 1% of bacteria have even been cultured, I would recommend revising language on this, especially since the species shown to require the Tat system are spread throughout the domain. It is remarkable that the model bacterium E. coli does not require it, and perhaps this investigation in Caulobacter can help us to understand variation in the essentiality of the Tat system.

We appreciate this comment and agree that we do not know what is prevalent in "most" bacteria. We have revised the manuscript to reflect what is currently known about some investigated bacteria and indicate that the question of essentiality has not been systematically explored (lines 70-78):

"While the Tat machinery is dispensable for viability in multiple model bacteria, including *Escherichia coli*, *Bacillus subtilis*, *Pseudomonas aeruginosa*, *Streptomyces coelicolor*, and *Yersinia pestis* and *Y. pseudotuberculosis* (Avican *et al.*, 2016; Bozue *et al.*, 2014; Jongbloed *et al.*, 2002; Ochsner *et al.*, 2002; Sargent *et al.*, 1999; Voulhoux *et al.*, 2001; Widdick *et al.*, 2006), it appears to be essential in a few species, such as *Mycobacterium tuberculosis* and *Bdellavibrio bacteriovorous* (Chang *et al.*, 2011; Palmer & Berks, 2012; Saint-Joanis *et al.*, 2006). Why the Tat system is essential in some bacteria but not others has not been explored in depth . . ."

As a follow-up to this point, we have added Figure 4 to show that PBP2 orthologs in a number of representative alpha-proteobacteria appear to contain Tat signal peptides.

2. E. coli encodes more Tat system components - TatABCDE - does Caulobacter only have TatABC (line75) or were these the only components identified as essential in the Tn-seq screen?

We appreciate the reviewer mentioning additional Tat system components. To our knowledge, *Caulobacter* encodes only the main three Tat components TatABC. There is a *tatD* gene that is annotated as a DNase in *Caulobacter*, and indeed *E. coli* TatD has also been reported to act mainly as a DNase rather than a core component of the Tat translocation complex (Chen *et al.*, 2014; Lindenstrauss *et al.*, 2010; Wexler *et al.*, 2000). *E. coli* has a TatA paralog called TatE (Eimer *et al.*, 2018), but it is expressed at lower levels and not required for Tat transport (Jack *et al.*, 2001; Sargent *et al.*, 1998). There is no gene annotated as TatE and no other known TatA paralogs in the *Caulobacter* genome. We did not investigate these genes and, for brevity, did not discuss them in the manuscript.

3. In lines 89-103 it is hard to know whether you are describing Pbp2 in E. coli or Caulobacter without looking up the references. It would be helpful to write more clearly in this section about what is known for Caulobacter vs. E. coli.

We have revised the Introduction section to try and make this clearer, indicating that much of the recent work on the elongasome and the cytoskeletal scaffold has been conducted in *E. coli*, but we also want to cite studies that have been done in other bacteria, such as *B. subtilis*. We aimed to balance both demands concisely.

4. Lines 125-133 describe the information shown in Figure 1A in the opposite order that they appear in the figure. I would make them match so that it is less confusing.

We have followed this helpful advice and arranged the panels in Figure 1A in the same order as the description in the text (lines 122-129).

5. Line 142 reports the average final OD610 of TatB to be 0.17 +/-0.01. In Figure 1B, it appears to be well below 0.05. Is there an explanation for this?

We thank the reviewer for this astute observation. It was a typo, and we missed the "0" after the decimal point. This sentence has been updated in the revised manuscript (lines 140-143):

> "In contrast, cultures cultivated with 0.1% glucose to repress expression of *tatA, tatB,* or *tatC* exhibited reduction in growth, reaching average final OD610 of 0.16±0.12, 0.02±0.01, or 0.29±0.02, respectively (Figure 1B, red curves)"

6. Line 151: Pbp2 is not a cytoskeletal protein; it is a PG-modifying periplasmic enzyme.

We appreciate this correction. We have updated the manuscript accordingly (lines 159-163):

“We therefore considered whether a protein that contributes to maintenance of cell shape may be impacted by the Tat complex. Because PBP2 transpeptidase activity is critical to PG remodeling, and it has a Tat recognition sequence in its N-terminus (Dilks *et al.*, 2003; Teufel *et al.*, 2022), it emerged as a candidate Tat substrate for further investigation.”

7. Line 153-154: since your pbp2 depletion strain does not deplete the entirety of Pbp2, just its transpeptidase domain, I would consider clarifying this in the notation for this strain in the figures and text.

Based on this feedback, we have now named the mutant *pbp2* allele as *pbp2*Δ_{TP} and explained the reason for using this construct (lines 164-169):

“To assess whether loss of PBP2 activity mimics loss of Tat activity, we first constructed a PBP2 depletion strain, with a mutant *pbp2* allele (*pbp2*Δ_{TP}) that fused its R267 codon to L621, thus deleting the endogenous region encoding the transpeptidase domain, and intact *pbp2* under the control of the *xylX* promoter (P_{xyl}-*pbp2*). As in another study (Wagner *et al.*, 2005), we were unable to generate a deletion of the complete *pbp2* coding sequence . . .”

We refer to the allele as such throughout the text and figures when appropriate.

8. In Figure 1, "C" is not labeled. I would consider breaking down these multiple components into i/ii/iii, etc, so you can discuss them more clearly in the text.

In response to the reviewers’ comments, we have changed the organization of Figure 1 and now labeled the multiple components separately to allow easier discussion in the text.

9. It could be helpful to do quantitative analysis on the morphologies of the different Tat and Pbp2 depletions using MicrobeJ (which you used in Fig 2 and 3 for demographs). This would help illustrate the degree to which the phenotypes differ.

We appreciate this suggestion and have now used MicrobeJ to calculate and report differences in cell “roundness” upon depletion of Tat components or PBP2 (Figure 1D). We also discuss these differences in the text (lines 188-204).

10. The quantification in Figure 2AB is never discussed in the MS.

We thank the reviewer for pointing out this oversight. We have modified how we assess the medial fluorescence profiles in Figures 2 and S3. We now calculate area under the curve (AUC), and this measurement is described in the Materials and Methods section (lines 424-431). Quantification results are discussed in the section “Tat complex is

required for PBP2 midcell localization upon osmotic upshift" (for example, lines 227-235).

11. Line 192 says the cells were grown in in M2G and immobilized on M2G pads whereas Figure 3 says "PYE culture -> PYE pad."

We thank the reviewer for noticing this error. Cells were grown in PYE liquid media and immobilized on PYE pads in Figures 3 and S4. This mistake has been corrected in the revised manuscript (lines 278-280):

"To verify that the Tat complex contributes to PBP2 subcellular localization even in the absence of osmotic shock, we imaged P_{van}-*mCherry-pbp2* cells grown in PYE media as above and then immobilized on PYE agarose pads."

References

Alcock, F., Stansfeld, P.J., Basit, H., Habersetzer, J., Baker, M.A., Palmer, T., Wallace, M.I., and Berks, B.C. (2016) Assembling the Tat protein translocase. *Elife* **5**.

Avican, U., Beckstette, M., Heroven, A.K., Lavander, M., Dersch, P., and Forsberg, A. (2016) Transcriptomic and Phenotypic Analysis Reveals New Functions for the Tat Pathway in *Yersinia pseudotuberculosis*. *J. Bacteriol.* **198**: 2876-2886.

Banzhaf, M., van den Berg van Saparoea, B., Terrak, M., Fraipont, C., Egan, A., Philippe, J., Zapun, A., Breukink, E., Nguyen-Disteche, M., den Blaauwen, T., and Vollmer, W. (2012) Cooperativity of peptidoglycan synthases active in bacterial cell elongation. *Mol Microbiol* **85**: 179-194.

Barnett, J.P., Eijlander, R.T., Kuipers, O.P., and Robinson, C. (2008) A minimal Tat system from a gram-positive organism: a bifunctional TatA subunit participates in discrete TatAC and TatA complexes. *J Biol Chem* **283**: 2534-2542.

Bernhardt, T.G., and de Boer, P.A. (2003) The *Escherichia coli* amidase AmiC is a periplasmic septal ring component exported via the twin-arginine transport pathway. *Mol Microbiol* **48**: 1171-1182.

Blaudeck, N., Kreutzenbeck, P., Muller, M., Sprenger, G.A., and Freudl, R. (2005) Isolation and characterization of bifunctional *Escherichia coli* TatA mutant proteins that allow efficient tat-dependent protein translocation in the absence of TatB. *J Biol Chem* **280**: 3426-3432.

Bozue, J., Cote, C.K., Chance, T., Kugelman, J., Kern, S.J., Kijek, T.K., Jenkins, A., Mou, S., Moody, K., Fritz, D., Robinson, C.G., Bell, T., and Worsham, P. (2014) A *Yersinia pestis tat* mutant is attenuated in bubonic and small-aerosol pneumonic challenge models of infection but not as attenuated by intranasal challenge. *PLoS One* **9**: e104524.

Bratton, B.P., Shaevitz, J.W., Gitai, Z., and Morgenstein, R.M. (2018) MreB polymers and curvature localization are enhanced by RodZ and predict *E. coli*'s cylindrical uniformity. *Nat Commun* **9**: 2797.

Brauer, A.M., Rogers, A.R., and Ellermeier, J.R. (2021) Twin-arginine translocation (Tat) mutants in *Salmonella enterica* serovar Typhimurium have increased susceptibility to cell wall targeting antibiotics. *FEMS microbes* **2**: xtab004.

Chang, C.Y., Hobley, L., Till, R., Capeness, M., Kanna, M., Burtt, W., Jagtap, P., Aizawa, S.I., and Sockett, R.E. (2011) The *Bdellovibrio bacteriovorus* twin-arginine transport system has roles in predatory and prey-independent growth. *Microbiology (Reading, England)* **157**: 3079-3093.

Chen, Y.C., Li, C.L., Hsiao, Y.Y., Duh, Y., and Yuan, H.S. (2014) Structure and function of TatD exonuclease in DNA repair. *Nucleic Acids Res* **42**: 10776-10785.

Christen, B., Abeliuk, E., Collier, J.M., Kalogeraki, V.S., Passarelli, B., Coller, J.A., Fero, M.J., McAdams, H.H., and Shapiro, L. (2011) The essential genome of a bacterium. *Mol Syst Biol* **7**: 528.

Dilks, K., Rose, R.W., Hartmann, E., and Pohlschröder, M. (2003) Prokaryotic utilization of the twin-arginine translocation pathway: a genomic survey. *J. Bacteriol.* **185**: 1478-1483.
Eimer, E., Kao, W.C., Frobel, J., Blummel, A.S., Hunte, C., and Muller, M. (2018) Unanticipated functional diversity among the TatA-type components of the Tat protein translocase. *Sci Rep* **8**: 1326.
Gallego-Parrilla, J.J., Severi, E., Chandra, G., and Palmer, T. (2024) Identification of novel tail-anchored membrane proteins integrated by the bacterial twin-arginine translocase. *Microbiology* **170**.
Garde, S., Chodisetti, P.K., and Reddy, M. (2021) Peptidoglycan: Structure, Synthesis, and Regulation. *EcoSal Plus* **9**.
Habersetzer, J., Moore, K., Cherry, J., Buchanan, G., Stansfeld, P.J., and Palmer, T. (2017) Substrate-triggered position switching of TatA and TatB during Tat transport in *Escherichia coli*. *Open Biol* **7**.
Ize, B., Stanley, N.R., Buchanan, G., and Palmer, T. (2003) Role of the *Escherichia coli* Tat pathway in outer membrane integrity. *Mol Microbiol* **48**: 1183-1193.
Jack, R.L., Sargent, F., Berks, B.C., Sawers, G., and Palmer, T. (2001) Constitutive expression of *Escherichia coli tat* genes indicates an important role for the twin-arginine translocase during aerobic and anaerobic growth. *J. Bacteriol.* **183**: 1801-1804.
Jongbloed, J.D., Antelmann, H., Hecker, M., Nijland, R., Bron, S., Airaksinen, U., Pries, F., Quax, W.J., van Dijl, J.M., and Braun, P.G. (2002) Selective contribution of the twin-arginine translocation pathway to protein secretion in *Bacillus subtilis*. *J Biol Chem* **277**: 44068-44078.
Jongbloed, J.D., Grieger, U., Antelmann, H., Hecker, M., Nijland, R., Bron, S., and van Dijl, J.M. (2004) Two minimal Tat translocases in Bacillus. *Mol Microbiol* **54**: 1319-1325.
Jongbloed, J.D., van der Ploeg, R., and van Dijl, J.M. (2006) Bifunctional TatA subunits in minimal Tat protein translocases. *Trends Microbiol* **14**: 2-4.
Judd, E.M., Comolli, L.R., Chen, J.C., Downing, K.H., Moerner, W.E., and McAdams, H.H. (2005) Distinct constrictive processes, separated in time and space, divide *Caulobacter* inner and outer membranes. *J. Bacteriol.* **187**: 6874-6882.
Keller, R., de Keyzer, J., Driessen, A.J., and Palmer, T. (2012) Co-operation between different targeting pathways during integration of a membrane protein. *J Cell Biol* **199**: 303-315.
Lindenstrauss, U., Matos, C.F., Graubner, W., Robinson, C., and Bruser, T. (2010) Malfolded recombinant Tat substrates are Tat-independently degraded in Escherichia coli. *FEBS Lett* **584**: 3644-3648.
Liu, X., Biboy, J., Consoli, E., Vollmer, W., and den Blaauwen, T. (2020) MreC and MreD balance the interaction between the elongasome proteins PBP2 and RodA. *PLoS Genet* **16**: e1009276.
Morgenstein, R.M., Bratton, B.P., Nguyen, J.P., Ouzounov, N., Shaevitz, J.W., and Gitai, Z. (2015) RodZ links MreB to cell wall synthesis to mediate MreB rotation and robust morphogenesis. *Proc. Natl. Acad. Sci. USA* **112**: 12510-12515.
Nygaard, R., Graham, C.L.B., Belcher Dufrisne, M., Colburn, J.D., Pepe, J., Hydorn, M.A., Corradi, S., Brown, C.M., Ashraf, K.U., Vickery, O.N., Briggs, N.S., Deering, J.J., Kloss, B., Botta, B., Clarke, O.B., Columbus, L., Dworkin, J., Stansfeld, P.J., Roper, D.I., and Mancia, F. (2023) Structural basis of peptidoglycan synthesis by *E. coli* RodA-PBP2 complex. *Nat Commun* **14**: 5151.
Ochsner, U.A., Snyder, A., Vasil, A.I., and Vasil, M.L. (2002) Effects of the twin-arginine translocase on secretion of virulence factors, stress response, and pathogenesis. *Proc. Natl. Acad. Sci. USA* **99**: 8312-8317.
Özbaykal, G., Wollrab, E., Simon, F., Vigouroux, A., Cordier, B., Aristov, A., Chaze, T., Matondo, M., and van Teeffelen, S. (2020) The transpeptidase PBP2 governs initial localization and activity of the major cell-wall synthesis machinery in E. coli. *Elife* **9**.
Palmer, T., and Berks, B.C. (2012) The twin-arginine translocation (Tat) protein export pathway. *Nat Rev Microbiol* **10**: 483-496.
Price, M.N., Wetmore, K.M., Waters, R.J., Callaghan, M., Ray, J., Liu, H., Kuehl, J.V., Melnyk, R.A., Lamson, J.S., Suh, Y., Carlson, H.K., Esquivel, Z., Sadeeshkumar, H., Chakraborty, R., Zane, G.M., Rubin, B.E., Wall, J.D., Visel, A., Bristow, J., Blow, M.J., Arkin, A.P., and Deutschbauer, A.M. (2018) Mutant phenotypes for thousands of bacterial genes of unknown function. *Nature* **557**: 503-509.
Rodrigue, A., Chanal, A., Beck, K., Muller, M., and Wu, L.F. (1999) Co-translocation of a periplasmic enzyme complex by a hitchhiker mechanism through the bacterial tat pathway. *J Biol Chem* **274**: 13223-13228.
Rohs, P.D.A., Buss, J., Sim, S.I., Squyres, G.R., Srisuknimit, V., Smith, M., Cho, H., Sjodt, M., Kruse, A.C., Garner, E.C., Walker, S., Kahne, D.E., and Bernhardt, T.G. (2018) A central role for PBP2 in

the activation of peptidoglycan polymerization by the bacterial cell elongation machinery. *PLoS Genet* **14**: e1007726.

Saint-Joanis, B., Demangel, C., Jackson, M., Brodin, P., Marsollier, L., Boshoff, H., and Cole, S.T. (2006) Inactivation of Rv2525c, a substrate of the twin arginine translocation (Tat) system of *Mycobacterium tuberculosis*, increases beta-lactam susceptibility and virulence. *J. Bacteriol.* **188**: 6669-6679.

Sargent, F., Bogsch, E.G., Stanley, N.R., Wexler, M., Robinson, C., Berks, B.C., and Palmer, T. (1998) Overlapping functions of components of a bacterial Sec-independent protein export pathway. *EMBO J* **17**: 3640-3650.

Sargent, F., Stanley, N.R., Berks, B.C., and Palmer, T. (1999) Sec-independent protein translocation in *Escherichia coli*. A distinct and pivotal role for the TatB protein. *J Biol Chem* **274**: 36073-36082.

Sauvage, E., Kerff, F., Terrak, M., Ayala, J.A., and Charlier, P. (2008) The penicillin-binding proteins: structure and role in peptidoglycan biosynthesis. *FEMS Microbiol Rev* **32**: 234-258.

Shlosman, I., Fivenson, E.M., Gilman, M.S.A., Sisley, T.A., Walker, S., Bernhardt, T.G., Kruse, A.C., and Loparo, J.J. (2023) Allosteric activation of cell wall synthesis during bacterial growth. *Nat Commun* **14**: 3439.

Spratt, B.G. (1975) Distinct penicillin binding proteins involved in the division, elongation, and shape of Escherichia coli K12. *Proc. Natl. Acad. Sci. USA* **72**: 2999-3003.

Teufel, F., Almagro Armenteros, J.J., Johansen, A.R., Gislason, M.H., Pihl, S.I., Tsirigos, K.D., Winther, O., Brunak, S., von Heijne, G., and Nielsen, H. (2022) SignalP 6.0 predicts all five types of signal peptides using protein language models. *Nat Biotechnol* **40**: 1023-1025.

Tooke, F.J., Babot, M., Chandra, G., Buchanan, G., and Palmer, T. (2017) A unifying mechanism for the biogenesis of membrane proteins co-operatively integrated by the Sec and Tat pathways. *Elife* **6**.

Voulhoux, R., Ball, G., Ize, B., Vasil, M.L., Lazdunski, A., Wu, L.F., and Filloux, A. (2001) Involvement of the twin-arginine translocation system in protein secretion via the type II pathway. *EMBO J* **20**: 6735-6741.

Wagner, J.K., Galvani, C.D., and Brun, Y.V. (2005) *Caulobacter crescentus* requires RodA and MreB for stalk synthesis and prevention of ectopic pole formation. *J. Bacteriol.* **187**: 544-553.

Wexler, M., Sargent, F., Jack, R.L., Stanley, N.R., Bogsch, E.G., Robinson, C., Berks, B.C., and Palmer, T. (2000) TatD is a cytoplasmic protein with DNase activity. No requirement for TatD family proteins in sec-independent protein export. *J Biol Chem* **275**: 16717-16722.

Widdick, D.A., Dilks, K., Chandra, G., Bottrill, A., Naldrett, M., Pohlschröder, M., and Palmer, T. (2006) The twin-arginine translocation pathway is a major route of protein export in *Streptomyces coelicolor*. *Proc. Natl. Acad. Sci. USA* **103**: 17927-17932.

Yen, M.R., Tseng, Y.H., Nguyen, E.H., Wu, L.F., and Saier, M.H., Jr. (2002) Sequence and phylogenetic analyses of the twin-arginine targeting (Tat) protein export system. *Arch Microbiol* **177**: 441-450.

RE: Manuscript #E24-12-0578R
TITLE: "Twin-arginine transport complex plays an essential role in Caulobacter cell shape and viability"

Dear Dr. Chen:

Thank you for your resubmission. Both reviewers now agree that this brief report contributes thoughtfully to our understanding of Tat function in alphaproteobacteria. Each has modest suggestions for improvement of the text. I recommend incorporating Reviewer 1's suggestion of clarifying use of "localization" and considering the points raised by Reviewer 2 and incorporating as you deem valuable.

Sincerely,
Erin Goley
Monitoring Editor
Molecular Biology of the Cell

Dear Dr. Chen,

The review of your manuscript, referenced above, is now complete. The Monitoring Editor has decided that your manuscript requires minor revisions before it can be published in Molecular Biology of the Cell, as described in the Monitoring Editor's decision letter above and the reviewer comments (if any) below.

A reminder: Please do not contact the Monitoring Editor directly regarding your manuscript. If you have any questions regarding the review process or the decision, please contact the MBoC Editorial Office (mboc@ascb.org).

When submitting your revision include a rebuttal letter that details, point-by-point, how the Monitoring Editor's and reviewers' comments have been addressed. (The file type for this letter must be "rebuttal letter"; do not include your response to the Monitoring Editor and reviewers in a "cover letter.") Please bear in mind that your rebuttal letter will be published with your paper if it is accepted, unless you have opted out of publishing the review history.

Authors are allowed 15 days to submit a revision. If this time period is inadequate, please contact us immediately at mboc@ascb.org.

In preparing your revised manuscript, please follow the instructions in the Information for Authors (www.molbiolcell.org/info-for-authors). In particular, to prepare for the possible acceptance of your revised manuscript, submit final, publication-quality figures with your revision as described.

To submit the rebuttal letter, revised version, and figures, please use this link (please enable cookies, or cut and paste URL):
Link Not Available

MBoC offers the option to publish your work with immediate open access. Open access can increase the discoverability and usability of your research. If you would like to publish your paper with immediate open access but did not select this option during initial submission, please contact us at mbc@ascb.org.

Authors of Articles and Brief Communications whose manuscripts have returned for minor revision ("revise only") are encouraged to create a short video abstract to accompany their article when it is published. These video abstracts, known as Science Sketches, are up to 2 minutes long and will be published on YouTube and then embedded in the article abstract. Science Sketch Editors on the MBoC Editorial Board will guide you as you prepare your video. Information about how to prepare and submit a video abstract is available at www.molbiolcell.org/science-sketches. Please contact mboc@ascb.org if you are interested in creating a Science Sketch.

Thank you for submitting your manuscript to MBoC. Please do not hesitate to contact this office if you have any questions.

Sign up for MBoC's new content alerts here https://tinyurl.com/3bcsyk3e

Sincerely,

Eric Baker
Journal Production Manager
MBoC Editorial Office

mbc@ascb.org

Reviewer #1 (Remarks to the Author):

Twin-arginine transport complex plays an essential role in Caulobacter cell shape and viability

TN Chong, DL Whitfield, M Panjalingam, N Pendar, JC Chen

The authors present a significantly more compelling demonstration that the twin-arginine translocation pathway (TAT) is essential in Caulobacter crescentus. The essentiality is confirmed via plasmid loss assay and depletion strain phenotypes. The authors have appropriate tempered their claim that the essentiality of TAT is due at least in part to a role in localizing PBP2 and provide logical alternative explanations for their observations in the text. The claim is supported by quantitative image analysis of cell shape and quantification of localization signals. Overall, this manuscript is much improved in both clarity of the text and the inclusion of additional data necessary to support the claims.

I have just one criticism for the authors to consider. Throughout the text the term localization is used interchangeably with translocation, secretion, or membrane insertion. I remain unconvinced that the localization of PBP2 to mid-cell upon osmotic upshift is directly dependent on the TAT but rather this may be driven by an association with divisome components. In this case, TAT is likely required for the membrane insertion and without that initial step, subcellular targeting to mid-cell cannot occur. Please consider carefully how the term localization is used throughout the text and where possible use the more accurate term (ie membrane insertion, translocation).

Reviewer #2 (Remarks to the Author):

It was great to see this paper again and to see that key confirmational experimental details have been added. The authors have provided sufficient experimental evidence that TatA is essential for targeting mCherry-PBP2 to the cell envelope by providing better images, quantification, and comparison to a known TatA cargo (ssTorA-dimer). They have also worked to better contextualize the study within current knowledge about the Rod system, of which PBP2 is a part. I congratulate the authors on an improved and interesting brief report. I only have minor suggestions that are not required for acceptance, especially since I understand that brief reports are limited to 20,000 characters:

Figure 4 is a nice addition to the paper and helps support its contention that the TAT-signal in PBP2 in Caulobacter is not species specific and what has been shown for Caulobacter has implications for other alphaproteobacteria. The authors may want to include in their Results/Discussion that the tree indicates that TAT-secretion of PBP2 is ancestral in the Alphaproteobacteria. This is a strong indication that future work in the Caulobacter system will have implications for understanding the essentiality of the TAT system for the whole class. Moreover, the fact that the TAT system is essential in Sinorhizobium when it no longer encodes PBP2 suggests other genetic networks that are impacted by TAT secretion, further complicating the question of what parameters lead to its essentiality.

The dispersed localization for PBP2 in the TatB complementation upon osmotic upshift (S3A) is definitely different from that for TatA and TatC. As for the reasoning discussed in lines 242-49, it doesn't seem logical suggest that expression of TatB from the Pxyl promoter is insufficient for operation of the Tat system because these cells look normal and wouldn't insufficient operation result in the morphological defects you see in Figure 1C? Are you maybe suggesting that expression of TatB is already so low from this promoter that even with xylose induction, it looks like depletion of TatC after 14-16 hours? Maybe clarify this section.

Line 272-5: It is true that we do not expect MreB and PBP1a to be dependent on Tat for localization. However, this experiment was suggested to test whether a third, Tat-secreted protein might be helping PBP2 or PBP1a to localize during osmotic upshift. The results of this experiment help clarify that the reason PBP2's localization is dependent on the Tat system is because it requires it for proper membrane insertion, not that another Tat cargo is required to help position the membrane-bound proteins identified by Hocking et al (2012), since PBP1a localization is not Tat-dependent.

In the section, "TatA is essential for localization of PBP2 and ssTorA" I would reorder the resuls you described to following this order: (1) ssTorA peripheral localization is dependent on Tat system, (2) PBP2 peripheral localization mimics that of ssTorA, (3) MreB and PBP1a do not.

Since Hocking et al. 2012 show that PBP2 and PBP1a show mid-cell localization during osmotic upshift but that MreB remains localized at mid-cell in absence of osmotic upshift, it seems like the PBP1a should be in the main figure 2 and MreB should be in the supplemental S3. That is, we're interested in showing that PBP1 (which does not have a Tat signal) still localizes normally during osmotic upshift, suggesting loss of PBP2 at mid-cell in the TatA depletion is due entirely to it not being inserted into the

membrane.

Dear Dr. Goley:

We thank you and the reviewers for responding positively to our revision of this brief report. We appreciate the reviewers' dedicated efforts and have replied to all their suggestions below. The text of the decision letter is shown in black Times New Roman font, while our replies are in blue Arial font.

Sincerely,
Joseph Chen

RE: Manuscript #E24-12-0578R
TITLE: "Twin-arginine transport complex plays an essential role in *Caulobacter* cell shape and viability"

Dear Dr. Chen:

Thank you for your resubmission. Both reviewers now agree that this brief report contributes thoughtfully to our understanding of Tat function in alphaproteobacteria. Each has modest suggestions for improvement of the text. I recommend incorporating Reviewer 1's suggestion of clarifying use of "localization" and considering the points raised by Reviewer 2 and incorporating as you deem valuable.

Sincerely,
Erin Goley
Monitoring Editor
Molecular Biology of the Cell

Reviewer #1 (Remarks to the Author):

Twin-arginine transport complex plays an essential role in *Caulobacter* cell shape and viability

TN Chong, DL Whitfield, M Panjalingam, N Pendar, JC Chen

The authors present a significantly more compelling demonstration that the twin-arginine translocation pathway (TAT) is essential in *Caulobacter crescentus*. The essentiality is confirmed via plasmid loss assay and depletion strain phenotypes. The authors have appropriate tempered their claim that the essentiality of TAT is due at least in part to a role in localizing PBP2 and provide logical alternative explanations for their observations in the text. The claim is supported

by quantitative image analysis of cell shape and quantification of localization signals. Overall, this manuscript is much improved in both clarity of the text and the inclusion of additional data necessary to support the claims.

I have just one criticism for the authors to consider. Throughout the text the term localization is used interchangeably with translocation, secretion, or membrane insertion. I remain unconvinced that the localization of PBP2 to mid-cell upon osmotic upshift is directly dependent on the TAT but rather this may be driven by an association with divisome components. In this case, TAT is likely required for the membrane insertion and without that initial step, subcellular targeting to mid-cell cannot occur. Please consider carefully how the term localization is used throughout the text and where possible use the more accurate term (ie membrane insertion, translocation).

We apologize for our sloppy use of the term localization throughout the text and agree completely with the reviewer. We believe that our *observations* via fluorescence microscopy show that localization of PBP2 to the cell envelope (and to the midcell upon osmotic upshift) depends on components of the Tat complex. Our most succinct *interpretation* of these results, combined with the leader sequence predictions, is that the Tat complex likely inserts PBP2 into the cell membrane. Without membrane translocation, PBP2 remains in the cytoplasm and cannot localize to the midcell upon osmotic upshift. Further biochemical work is needed to verify conclusively that the Tat complex directly inserts PBP2 into the membrane. We have now tried to make this interpretation of the microscopy results more explicit throughout the text. For example, we added the following sentence (lines 236-239):

"Along with the presence of a Tat leader sequence in PBP2 (Dilks *et al.*, 2003; Teufel *et al.*, 2022), these localization results suggest that the Tat complex is necessary for membrane insertion of PBP2 in *Caulobacter*, allowing subsequent recruitment to the division site upon osmotic upshift."

Reviewer #2 (Remarks to the Author):

It was great to see this paper again and to see that key confirmational experimental details have been added. The authors have provided sufficient experimental evidence that TatA is essential for targeting mCherry-PBP2 to the cell envelope by providing better images, quantification, and comparison to a known TatA cargo (ssTorA-dimer). They have also worked to better contextualize the study within current knowledge about the Rod system, of which PBP2 is a part. I congratulate the authors on an improved and interesting brief report. I only have minor suggestions that are not required for acceptance, especially since I understand that brief reports are limited to 20,000 characters:

Figure 4 is a nice addition to the paper and helps support its contention that the TAT-signal in PBP2 in *Caulobacter* is not species specific and what has been shown for *Caulobacter* has implications for other alphaproteobacteria. The authors may want to include in their Results/Discussion that the tree indicates that TAT-secretion of PBP2 is ancestral in the *Alphaproteobacteria*. This is a strong indication that future work in the *Caulobacter* system will

have implications for understanding the essentiality of the TAT system for the whole class. Moreover, the fact that the TAT system is essential in *Sinorhizobium* when it no longer encodes PBP2 suggests other genetic networks that are impacted by TAT secretion, further complicating the question of what parameters lead to its essentiality.

We thank the reviewer for helping us emphasize the significance of the study and have added the suggested changes to the discussion of Figure 4.

"Thus, because Tat-dependent export of PBP2 appears ancestral in *Alphaproteobacteria*, future work probing the essentiality of this process in *Caulobacter* will have implications for the entire class. A notable exception is the *Rhizobiales/Hyphomicrobiales* group, which lacks PBP2 and MreB (Brown *et al.*, 2012; Cameron *et al.*, 2014; Williams *et al.*, 2021) and includes both *S. meliloti* and *A. tumefaciens*, in which the Tat system is essential and non-essential, respectively. Investigation in those models may reveal additional genetic circuits and parameters that dictate whether the Tat pathway is essential for viability." (lines 362-369)

The dispersed localization for PBP2 in the TatB complementation upon osmotic upshift (S3A) is definitely different from that for TatA and TatC. As for the reasoning discussed in lines 242-49, it doesn't seem logical suggest that expression of TatB from the P_{xyl} promoter is insufficient for operation of the Tat system because these cells look normal and wouldn't insufficient operation result in the morphological defects you see in Figure 1C? Are you maybe suggesting that expression of TatB is already so low from this promoter that even with xylose induction, it looks like depletion of TatC after 14-16 hours? Maybe clarify this section.

We appreciate this suggestion to be more precise in our explanation of why mCherry-PBP2 exhibits dispersed localization upon osmotic upshift in the TatB depletion strain, even when grown with xylose. We have added a sentence to indicate that the membrane insertion of mCherry-PBP2 under that condition may be less efficient than that in wild type yet still sufficient to maintain cell shape, perhaps similar to the TatC depletion strain after growth in glucose for 14 – 16 hours. Moreover, a higher percentage of the fusion protein the cytoplasm can obscure the fluorescence signal of the membrane-bound portion, as seen in Figure 3 for ssTorA-dimer2: a shift in the ratio of cytoplasmic versus exported dimer2 can affect the readout.

"Even when P_{xyl}-*tatB* is induced, a significant fraction of mCherry-PBP2 may remain in the cytoplasm, and their fluorescence would obscure the midcell localization signal, yet there would be sufficient membrane-inserted PBP2 to maintain cell shape. As the midcell signal is already low in TatB-replete cells, depletion of TatB leads to only a minute reduction in that signal." (lines 248-253)

"For example, growth of the TatB depletion strain in xylose may only allow translocation at an efficiency comparable to that of the TatC depletion strain after shift to glucose for more than 12 hours, resulting in limited midcell localization of mCherry-PBP2 in cells where TatB should be replete (Figure S3, A and B) and rapid growth arrest upon shift to glucose (Figure 1B), in contrast to observations with the TatC depletion strain." (lines 265-270)

Line 272-5: It is true that we do not expect MreB and PBP1a to be dependent on Tat for localization. However, this experiment was suggested to test whether a third, Tat-secreted protein might be helping PBP2 or PBP1a to localize during osmotic upshift. The results of this experiment help clarify that the reason PBP2's localization is dependent on the Tat system is because it requires it for proper membrane insertion, not that another Tat cargo is required to help position the membrane-bound proteins identified by Hocking et al (2012), since PBP1a localization is not Tat-dependent.

We appreciate this clarification and have added this interpretation (lines 286-290):

"Together, these results indicate that, unlike PBP2, PBP1a and MreB do not depend on the Tat complex for subcellular localization. Instead of another Tat cargo that recruits membrane proteins such as PBP2 and PBP1a to the division site upon osmotic shock, PBP2's dependence is more likely due to its reliance on the Tat apparatus for membrane insertion."

In the section, "TatA is essential for localization of PBP2 and ssTorA" I would reorder the results you described to following this order: (1) ssTorA peripheral localization is dependent on Tat system, (2) PBP2 peripheral localization mimics that of ssTorA, (3) MreB and PBP1a do not.

We thank the reviewer for this suggestion and have changed the order of how these results are presented.

Since Hocking et al. 2012 show that PBP2 and PBP1a show mid-cell localization during osmotic upshift but that MreB remains localized at mid-cell in absence of osmotic upshift, it seems like the PBP1a should be in the main figure 2 and MreB should be in the supplemental S3. That is, we're interested in showing that PBP1 (which does not have a Tat signal) still localizes normally during osmotic upshift, suggesting loss of PBP2 at mid-cell in the TatA depletion is due entirely to it not being inserted into the membrane.

We appreciate this thoughtful suggestion. We were initially concerned that midcell localization of PBP1a may not be as convincing or definitive as that of MreB. However, based on the reviewers' feedback, we have now changed the presentation of Figures 2 and S3.

RE: Manuscript #E24-12-0578RR
TITLE: "Twin-arginine transport complex plays an essential role in Caulobacter cell shape and viability"

Dear Dr. Chen:

I am pleased to accept your manuscript for publication in Molecular Biology of the Cell.

Thank you for addressing each of the reviewers' remaining suggestions. Your manuscript is a nice contribution to our understanding of secretion and morphogenesis in alphaproteobacteria.

Sincerely,
Erin Goley
Monitoring Editor
Molecular Biology of the Cell

Dear Dr. Chen:

Congratulations on the acceptance of your manuscript! Thank you for publishing your work in Molecular Biology of the Cell (MBoC).

Within 10 days, an unedited PDF of your manuscript will be published on MBoC in Press, an early-release journal version. The date your manuscript appears on this site is the official publication date.

Your copyedited and typeset manuscript will be scheduled for publication in the next available issue of MBoC. Once your paper is ready for review, our production team will notify you. In the summer of 2024, our production provider will introduce an online proofing solution, which will streamline the process of checking and validating author changes. This will result in improved typesetting quality, enhanced digital content, and a faster overall process.

We invite you to submit images related to your accepted manuscript to be considered for the journal cover.
Cover images must contain color, TIF Format
Size image at 7.4" wide x 5.85" deep
RGB and CMYK color (please submit two separate files, one in each format)
300 dpi resolution

Please also send a 1-paragraph description of the image that a general audience could understand. Include with the caption the name and institution of the individual(s) the image should be attributed to. Please provide this description in a Word file. You can send the file via email or by your favorite file transfer service to mboc@ascb.org.

Authors of Articles and Brief Communications are also encouraged to create a short video abstract to accompany their article when it is published. These video abstracts, known as Science Sketches, are up to 2 minutes long and will be published on YouTube and then embedded in the article abstract. Science Sketch Editors on the MBoC Editorial Board will guide you as you prepare your video. Information about how to prepare and submit a video abstract is available at www.molbiolcell.org/science-sketches. Please contact mboc@ascb.org if you are interested in creating a Science Sketch.

We look forward to publishing your paper in MBoC.
Sign up for MBoC's new content alerts here https://tinyurl.com/3bcsyk3e

Sincerely,

Eric Baker
Journal Production Manager
MBoC Editorial Office
mbc@ascb.org
